# Enhancement of Photorefraction in Vanadium-Doped Lithium Niobate through Iron and Zirconium Co-Doping

**DOI:** 10.3390/ma12193143

**Published:** 2019-09-26

**Authors:** Shahzad Saeed, Hongde Liu, Liyun Xue, Dahuai Zheng, Shiguo Liu, Shaolin Chen, Yongfa Kong, Romano Rupp, Jingjun Xu

**Affiliations:** 1 The MOE Key Laboratory of Weak-Light Nonlinear Photonics, School of Physics and TEDA Institute of Applied Physics, Nankai University, Tianjin 300071, China; shehzadsaeed2003@yahoo.com (S.S.); 1120150044@mail.nankai.edu.cn (L.X.); dhzheng@nankai.edu.cn (D.Z.); nkliusg@nankai.edu.cn (S.L.); chenshaolin@nankai.edu.cn (S.C.); jjxu@nankai.edu.cn (J.X.); 2 Faculty of Physics, Vienna University, A-1090 Wien, Austria; romano.rupp@univie.ac.at; 3 Department of Complex Matter, Jozef Stefan Institute, Jamova 39, SI-1000 Ljubljana, Slovenia

**Keywords:** lithium niobate, photorefractive properties, optical storage materials, vanadium, zirconium and iron co-doped

## Abstract

A series of mono-, double-, and tri-doped LiNbO_3_ crystals with vanadium were grown by Czochralski method, and their photorefractive properties were investigated. The response time for 0.1 mol% vanadium, 4.0 mol% zirconium, and 0.03 wt.% iron co-doped lithium niobate crystal at 488 nm was shortened to 0.53 s, which is three orders of magnitude shorter than the mono-iron-doped lithium niobate, with a maintained high diffraction efficiency of 57% and an excellent sensitivity of 9.2 cm/J. The Ultraviolet-visible (UV-Vis) and OH^−^ absorption spectra were studied for all crystals tested. The defect structure is discussed, and a defect energy level diagram is proposed. The results show that vanadium, zirconium, and iron co-doped lithium niobate crystals with fast response and a moderately large diffraction efficiency can become another good candidate material for 3D-holographic storage and dynamic holography applications.

## 1. Introduction

Lithium niobate (LN), due to its excellent properties, has a variety of applications from waveguides and resonators to integrated optical devices and optical modulators to holographic storage [1,2,3,4,5,6,7,8]. Holographic storage has garnered a considerable research interest due to its potential future applications and is studied by many researchers from an extended period. The superior competence of holographic storage involves its high storage capacity and faster data transfer rate [7,8,9,10,11]. Recently, much research is also directed toward the photorefractive polymers and other organic materials, due to their faster photorefractive (PR) responses and high sensitivities for holographic storage applications. Some researchers have started commercial applications, such as in image holography, etc., but they also have some practical hurdles that remain due to their size and applied high voltages [10,11,12]. Despite that, LN crystal is still considered the most popular material media for 3D-holographic storage devices and dynamic holography applications [5,10,13]. Up to now, various mono-, double-, and tri-doped LN crystals with dopants, such as Fe, Mn, V, Bi, Mo, In, Zn, Zr, Mg, etc., were studied for PR properties enhancement [13,14,15,16,17,18,19,20]. Fe doping in LN is the major candidate and is extensively studied by a large number of the researchers; the long response time, scattering, and volatility of these crystals are still in the phase of development [13,14,15,16,21]. To realize a nonvolatile readout, several methods have been developed so far, i.e., thermal fixing, electrical fixing, and two-step recording [14,22]. Though, up to now, there is good improvement in the response speed of the various doped LN crystals [23,24,25,26], much research is needed to get a practical fast responsive material with a high diffraction efficiency. Recently it was found that vanadium doping in LN elicited shortening of the response time [18,19,26,27]. Moreover, the optical damage resistance (ODR) ions, i.e., Mg, Zr, In, etc., in co-doping with PR-ions, i.e., Fe, Mo, Bi, V, etc., can also help minimize the unwanted slow intrinsic defect traps, by improving response speed [23,24,25,26,28].

In this paper, we have grown several various double- and triple-doped LN crystals, such as vanadium and zirconium (LN:V,Zr), and vanadium, zirconium, and iron (LN:V,Zr,Fe), to investigate how these various types of co-doping (vanadium, zirconium, and iron) with different valences in LN affect its photorefractive properties.

## 2. Materials and Methods

### 2.1. Samples Preparation

Using the ordinary Czochralski method, we have successfully grown several LN:V,Zr; LN:V,Zr,Fe crystals in air along the c-axis at fixed vanadium concentration at 0.1 mol% and iron concentration at 0.03 wt.% in all crystals, whereas the concentration of zirconium of varied (2.0, 3.0, and 4.0 mol%). Also, we grew the congruent LN (CLN), 0.1 mol% vanadium-doped LN (LN:V_0.1_) and vanadium and iron co-doped LN (LN:V,Fe_0.03_) for comparison and characterization measurements. These crystals were labeled as LN:V,Zr_2.0_,Fe (LN1), LN:V,Zr_3.0_,Fe (LN2), LN:V,Zr_4.0_,Fe (LN3), LN:V,Zr_2.0_ (LN4), LN:V,Zr_3.0_ (LN5), LN:V,Zr_4.0_ (LN6), LN:V,Fe_0.03_ (LN7), and LN:V_0.1_ (LN8), respectively, and their different composition ratio is shown in Table 1. For CLN, we selected the congruent composition of [Li]/[Nb] = 48.38/51.62. The diameter of the crystals after completion of the growth process was ~30 mm, with a length of 35 mm approximatley along the z-axis. After the growth process, we annealed and polarized the crystals in a furnace, with the uniform temperature kept at 1120 °C for 20 h and Direct current of 15 mA given to the crystals for 15 min. Then the crystals were cut along y-faces into the plates of thickness size of 1.0 mm and 3.0 mm. The plate’s dimensional sizes were 30 mm in length and 20 mm in width along the z–x direction. Finally, we polished these crystal plates up to optical grade for experimental and characterization measurements. The 3.0 mm crystals were used for the measurement of PR properties, and the 1.0 mm crystals were used for characterization measurement.

### 2.2. Measurements

The PR properties of all the crystals were studied by two-wave coupling method at a laser wavelength of 488 nm (Ar^+^ laser) and 532 nm (CW frequency-doubled solid-state laser). The 3.0 mm y-oriented crystal plates were illuminated with two equal-intensity laser horizontal-polarized beams, with a 30° crossing angle and the value of net total intensity for beams (signal + reference) used at both wavelengths was 500 mW/cm^2^. The PR properties were measured and calculated, i.e., diffraction efficiency *η* = *I*_d_/(*I*_d_ + *I*_t_), and “*I*_t_” and “*I*_d_” are the readout beam transmitted and diffracted light intensity; PR-response time constant, *τ*_r_, and saturated diffraction efficiency, *η*_s_, satisfy the formula $\;\eta_{t}=\eta_{s}{\left(1-e^{-t/\tau_{r}}\right)}^{2}$; change of the refractive index Δ*n* is from the formula *η* = sin^2^(πdΔn/λcosθ), where *d* is the grating thickness of the sample, *λ* is free-space wavelength, and “*θ*” is the Bragg angle; the PR-sensitivity$\;S={\left(\left(\partial \sqrt{\eta }\right)/\partial t\right)}_{t=0}/Il\approx \frac{1}{Il}\frac{\sqrt{\eta_{s}}}{\tau }$, “*l*” is the thickness of crystal plates and “*I*” is total recording light intensity [8]. We used the transmission geometry for all the performed experiments, where two recording beams were incident on the same face of the storage medium. For recording and reading process, the extraordinary polarization was used because the recording speed and diffraction efficiency are larger than that of ordinary polarization for transmission geometry. To measure *η*, one of the writing beams was blocked momentarily by an electronic shutter at different regular intervals of time. Distinct positions of crystal were selected for the measurements in order to check nonuniformity in the results. The results achieved were similar, with little error margins for each position, which further confirms the validity of the results and shows that there is no variation in the composition of the crystals. The diffracted beam was continuously monitored with an optical power meter.

The UV-Vis absorption spectra were measured by a UV-4100 spectrophotometer (Hitachi Science and Technology, Tokyo, Japan) with a range of 300 to 800 nm and a resolution of 1 nm. A MAGNA-560 Fourier-Transform Infrared Spectrometer (Thermo Nicolet Corporation, Madison, WI, USA) was used for infrared (IR) spectroscopy with a range of 400 to 4000 cm^−1^ and a resolution of 2 cm^−1^. All of the samples were 1.0 mm y-oriented plates for the spectrum measurements, and the incoming light was unpolarized.

## 3. Results

### 3.1. The Photorefractive Properties

**Figure 1:**
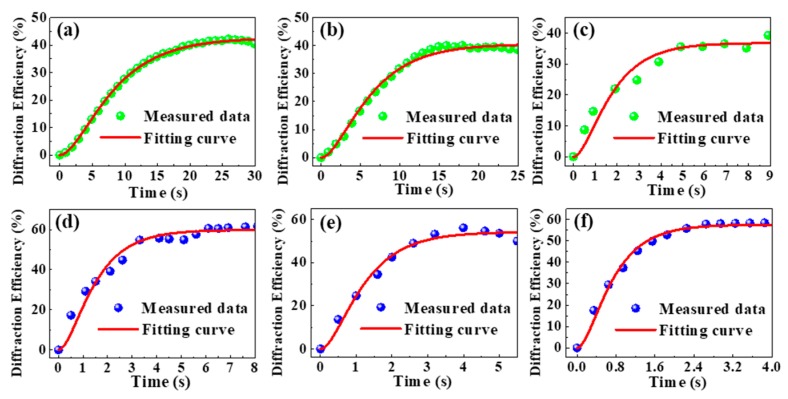
Evolution of diffraction efficiency with time for LN:V,Zr,Fe crystals: (**a**) LN1, (**b**) LN2, and (**c**) LN3 at 532 nm, respectively, and (**d**) LN1, (**e**) LN2, and (**f**) LN3 at 488 nm, respectively.

**Figure 2:**
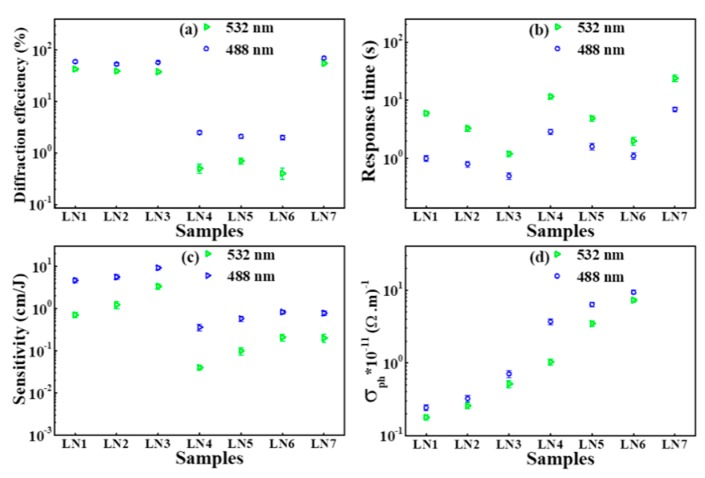
The measured (**a**) diffraction efficiency, (**b**) PR response time, (**c**) sensitivity, and (**d**) photoconductivity (σ_ph_) of LN-doped (LN1–LN7) at 532 nm and 488 nm, respectively.

Figure 1 shows the evolution of diffraction efficiency (*η*) with time for LN:V,Zr,Fe (LN1–LN3) crystals at 532 nm and 488 nm. From the measured data of the time variance diffraction efficiency, the PR properties, such as the saturated diffraction efficiency (*η_s_*), response time (τ_r_), and sensitivity (S), were calculated for LN1–LN7 crystals and are shown in Figure 2. From Figure 2a, we can see that the saturated diffraction efficiency (*η_s_*) for LN1, LN2, and LN3 crystals reaches ~57% and ~40% at 488 nm and 532 nm, respectively, which are one order of magnitude higher than LN4, LN5, and LN6. The response time for LN1, LN2 and LN3 is shortened, and sensitivity is increased with increased zirconium concentration. The response speed of LN3 (LN:V,Zr_4.0_,Fe) is fast compared to others and reaches to 0.53 s at 488 nm, which is two order magnitude shorter than LN7 and one order magnitude shorter than LN4, LN5 and LN6. The sensitivity of LN3 crystal can reach 9.2 cm/J, which is higher than other LN crystals under the same conditions.

Moreover, the saturated diffraction efficiency (*η_s_*) of LN,V,Zr (LN4–LN6) crystals is lower than other LN1, LN2, LN3, and LN7 crystals and is similar to LN8 and CLN. However, their response speed and sensitivity were also improved with increasing zirconium concentration, analogous to the LN1, LN2, and LN3 crystals. The response time for LN6 (LN:V,Zr_4.0_) is 1.1 s and 2.0 s at 488 nm and 532 nm, respectively. It is not as short as mono vanadium-doped LN (LN8), but it is still two orders shorter than CLN and LN:Fe and is one order shorter compared to LN:Mg,Fe, and is also a fraction shorter than LN7 at 532 nm and 488 nm, respectively [23,24,25,26,28]. The LN7 (LN:V,Fe) crystal enjoys higher diffraction efficiency at 532 nm and 488 nm compared to LN1-LN6, LN8, CLN, LN:Zr,Fe, and LN:Mg,Fe. Nevertheless, its response speed of 7 s and 24 s at 488 nm and 532 nm is not as short as LN1–LN6, LN8, and LN:Zr,Fe, but is still one and two orders of magnitude shorter than LN:Mg,Fe and CLN in the same wavelength region, respectively. For comparison, we have selected well known co-doped LN crystals and their PR properties are shown in Table 2.

The photoconductivity was calculated from the behavior of the erasure curve for LN1–LN6 crystals by the equation σ_ph_ = εε_0_/τ_e_, where ε_0_ is the dielectric constant for vacuum, τ_e_ denotes time constant, and ε = 28 is the LN dielectric constant [29]. The experimental data of the erasure curve was exploited through the double exponential fitting function η = η_1_ exp(−2t/τ_1_) + η_2_ exp(−2t/τ_2_). From Figure 2d, we can see that the photoconductivity depends linearly on zirconium concentration and enhanced with increasing zirconium concentration for LN1–LN3 and LN4–LN6 at 488 nm and 532 nm, respectively. However, the photoconductivity is higher for LN4–LN6 compared to LN1–LN3.

### 3.2. The UV–Vis Absorption Spectra

**Figure 3:**
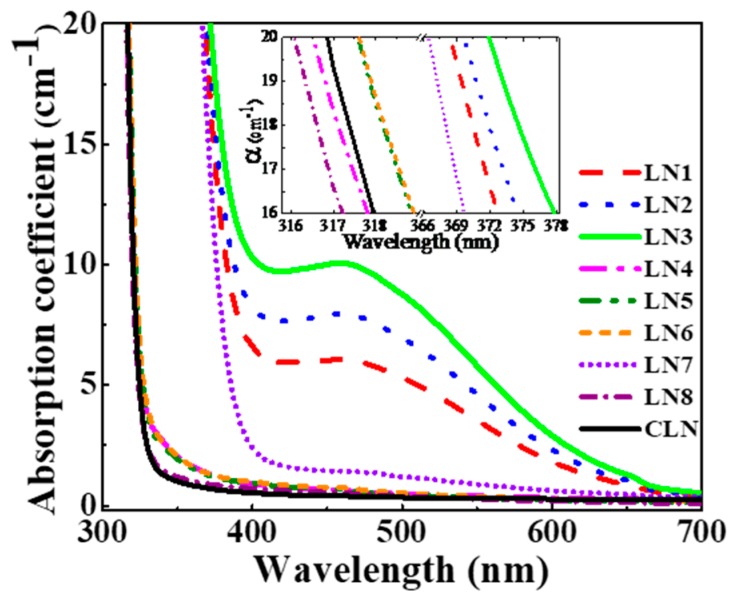
UV–Vis absorption spectra for all the prepared samples of LN1–LN8 and CLN; the inset shows the absorption edges for each crystal at 20 cm^−1^.

The UV–Vis absorption spectra were measured for all crystals; so as to know about the defects and various concentration and doping-related changes in it, the absorption spectra are shown in Figure 3. The constituent of Li–O, and Nb–O chemical bonds governs the optical and many other physical properties in LN, i.e., optical absorption, bandgap, index of refraction, etc. [20,30,31,32,33]. Further, the change in the defects states of Nb_Li_ antisites is mostly responsible for shifts in the absorption edges. For all prepared samples, the absorption edges at 20 cm^−1^ are in the inset of Figure 3. We can see that the absorption edges for LN1, LN2, and LN3 are located at 368.8 nm, 370.0 nm, and 372.0 nm, respectively; LN4, LN5, and LN6 at 316.5 nm, 317.6 nm, and 317.6 nm, respectively; and LN7, LN8, and CLN at 366.3 nm, 316.1 nm, and 316.9 nm, respectively.

The absorption edges are violet-shifted for LN4 and LN8, whereas that of LN1–LN3, LN5–LN7 is red-shifted, compared to CLN. Further, the absorption edge of LN7 is red-shifted compared to LN4, LN5, and LN6 and violet-shifted compared to LN1, LN2, and LN3. We observe that the absorption edges start moving toward the red, with increasing zirconium concentration for LN1–LN3 and LN4–LN6 crystals, respectively. As we know that the Zr-doping concentration in our crystals is already above its doping concentration threshold of 2.0 mol%, the movement of absorption edges toward the red with increase zirconium concentration above 2 mol% is in confirmation with other research reports [34,35].

For LN1, LN2, and LN3, there is a broad absorption band slightly initiated from 700 to 380 nm. This absorption band is related to the concentration of ${\mathrm{Fe}}_{\mathrm{Li}}^{2+}$ ions according to the authors of [15] and becomes more in-depth with increasing zirconium concentration. This shows the increase in ${\mathrm{Fe}}_{\mathrm{Li}}^{2+}$ donor impurities with increasing zirconium concentration, which have normally occurring peaks in this range of wavelength for absorption spectra. The increase of Fe^2+^ concentration is consistent with the proportional enhancement of photoconductivity and response speed mentioned above, which give an evidence for ${\mathrm{Fe}}_{\mathrm{Li}}^{2+}$ ions as donor center.

### 3.3. The OH^−^ Absorption Spectra

**Figure 4:**
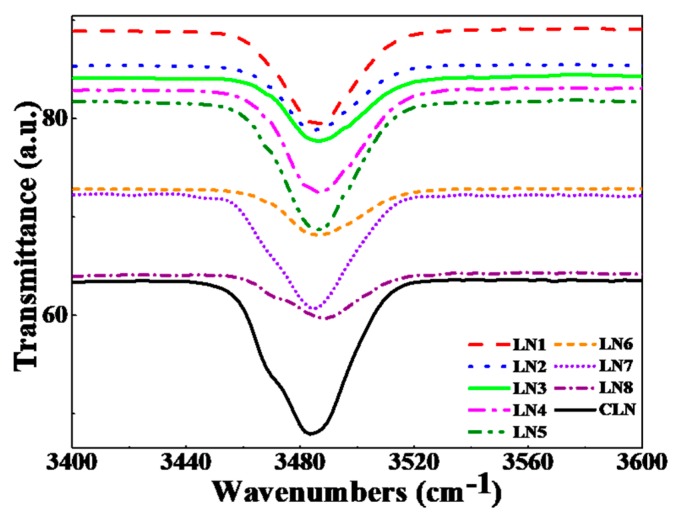
OH^−^ absorption spectra for LN1-LN8 and CLN (top to bottom) crystals.

Figure 4 shows the OH^−^ absorption spectrum in the range of 3400 to 3600 cm^−1^, which helps us to observe the structural changes of LN crystals with various doping [13,20,36,37]. From Figure 4, we can see that the OH^−^ absorption peaks for LN1, LN2, LN4, LN5, and LN7 appear at 3485 cm^−1^, LN3 and LN6 appear at 3487 cm^−1^, and LN8 and CLN appear at 3489 cm^−1^ and 3483 cm^−1^. We can see that the OH absorption peaks slightly move toward high wavenumber with increased zirconium concentration for LN4 and LN8 compared to LN2, LN3 and LN6, LN7, respectively.

It is reported that OH^−^ absorption gives peaks in the range of 3400 to 3600 cm^−1^ for LN crystals that usually correspond to the Nb_Li_ antisites’ defect. The peaks will start moving to lower wavenumbers with a reduction of these antisites’ defect states. The peaks movement toward the high wavenumbers varies according to the valence of the doped ODR ion, and the higher the valence, the lower is the hydroxyl vibration frequency movement to higher wavenumber [13,36,37]. Based on the above results, it verifies that the intrinsic slow defect traps were further reduced with increasing zirconium concentration that results in further enhancement of response speed. Besides, it is well known that there is a 3507 cm^−1^ peak corresponding to the vibration of ${\mathrm{Fe}}_{\mathrm{Nb}}^{3+}–{\mathrm{OH}}^{-}$ in LN when the ODR ions concentration beyond its threshold, which means ${\mathrm{Fe}}_{\mathrm{Li}}^{3+}$ ions move into Nb-sites and cause an abrupt increase of its capture cross section for electrons. There is no 3507 cm^−1^ peak existing in Figure 3, which suggests that the lattice locations of ${\mathrm{Fe}}^{3+}$ ions are still at Li-site whenever the ZrO_2_ concentration goes above the threshold.

## 4. Discussion

Vanadium-doped LN (LN:V), in comparison to other mono-doped LN, exhibits a fast response time of 570 ms at 532 nm, but its diffraction efficiency is only ~1%, which is very low for practical application [18]. From the above results, it is evident that the zirconium and iron co-doping has improved the diffraction efficiency for LN:V from 1% to 40% and 57% at 532 nm and 488 nm, respectively. Its response speed is also somewhat improved compared to that of LN:V [18], and several orders of magnitude enhanced compared to CLN, LN:Fe [21,25,28] and other co-doped LN crystals. The PR properties of LN:V,Zr,Fe are better than LN:Fe,Mg [28], LN:Zr,Fe [15], and LN:Mn,Zr,Fe [16], and are comparable to that of LN:Bi,Mg [25]. LN:Bi,Mg has comparatively fast response speed but has low diffraction efficiency of ~18%. Therefore, the LN:V,Zr,Fe crystal are more suitable for holography storage and dynamic holographic applications.

**Figure 5:**
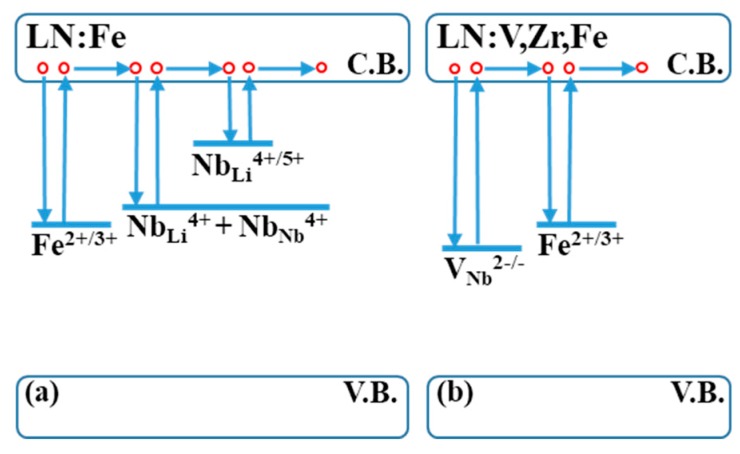
Schematic energy level diagram for (**a**) LN:Fe and (**b**) LN:V,Zr,Fe with intrinsic and extrinsic defects.

As is well known, the enhancement of PR properties is related to the change in defect structure in LN. Regarding the defect structure, congruent LN is studied well, and from the “Li vacancy model”, many types of intrinsic defect states, i.e., (a) electron-bound polarons $\left({\mathrm{Nb}}_{\mathrm{Li}}^{4+}\right)$ located at Li sites, (b) electron-free polarons ${(\mathrm{Nb}}_{\mathrm{Nb}}^{4+})$ located at Nb ions, (c) bipolarons ${(\mathrm{Nb}}_{\mathrm{Nb}}^{4+}$ + ${\mathrm{Nb}}_{\mathrm{Li}}^{4+})$ as a combination of a bound and free electron polaron, some oxygen vacancies and antisites, etc., were recognized due to the naturally occurring Li vacancies during crystal growth. The LN:Fe crystal is a common candidate material for volatile and nonvolatile holographic data storage, although it also contains these intrinsic defects as mentioned for CLN (Figure 5a) [38,39]. To solve the issue of naturally occurring Li vacancies, stoichiometric LN (SLN) and various doped LN crystal have been grown, such as SLN:Fe, LN:Mg,Fe, LN:Zr,Fe, etc.; however, it is reported that doping of low valence dopants, i.e., Mg^2+^, Zn^2+^, Sc^3+^, or In^3+^, above a specific threshold concentration into LN:Fe can make a significant enhancement in the photorefractive response speed and make ${\mathrm{Nb}}_{\mathrm{Li}}^{4+}\;$push back to normal Nb-sites by these doping ions [13,14,16,20,28,40]. However, mostly, these dopants are divalent (+2) or trivalent (+3), and also prefer to occupy regular lithium vacancy sites. As Fe^2+/3+^ also prefers to occupy normal lithium vacancy sites, the abundance of these low valance doping ions occupying Li vacancy sites can disrupt the occupation of Fe ions on regular lithium vacancy sites; unfortunately, because of this, Fe^3+^ and part of the Fe^2+^ ions are thereby pushed into Nb-sites, which causes these Fe^2+/3+^ ions to lose their function as photorefractive centers. Thus the photorefractive diffraction efficiency also decreases, and there is no noticeable increase in photorefractive sensitivity. For example, the LN:Mg,Fe [28] have improved the response speed but have reduced the diffraction efficiency also. Hence it is necessary to introduce dopants of high valence with the LN:Fe to maintain its regular occupying sites and to further enhance its photorefractive properties also.

Vanadium, as a high valence dopant, is introduced into LN7 crystal. However, LN7 shows a high diffraction efficiency, but it is slow, with a response time of ten seconds. For the enhancement of response speed, zirconium is doped into LN crystals as the 3rd dopant. It is believed that the doping properties are related to the essential characteristics of the dopants. For example, the tetravalent Zr^4+^ ion may be more stable than the Mg^2+^ ion, due to the similar ionic radius and valence state to the Nb^5+^ ion, and because co-doping with Zr eliminates undesirable intrinsic traps around its low doping threshold of 2.0 mol%, which strongly enhances the charge transition speed. As Kong et al. improved the response speed by doping tetravalent Zr^4+^ into LN:Fe and LN:Mn,Fe [15,16]. Therefore, further vanadium doping seems a well-suited and more stable doping substitution in this respect and rivals Nb, which also has a low doping threshold compared to other high-valent dopants for PR propertu enhancement, has a higher valence (+5) than Zr^4^, and is quite similar in electronegativity (1.63 eV) and atomic resemblance to Nb^5+^. Therefore, the vanadium ions occupying the Nb site are preferred to Zr and Fe ions in LN:V,Zr,Fe.

Dong et al. reported that vanadium exhibits three valences, V^3+^, V^4+^, and V^5+^ ions in LN:V, among which V^3+^ and V^4+^ substitute ${\mathrm{Nb}}_{\mathrm{Li}}^{4+}$, forming new defects of $V_{\mathrm{Li}}^{2+}$ and $V_{\mathrm{Li}}^{3+}$, V^5+^ will partially occupy Nb_Li_ and Nb sites forming $V_{\mathrm{Li}}^{4+}$ and a neutral state of $V_{\mathrm{Nb}}^{0}$ [18,19]. It is thought that $V_{\mathrm{Li}}^{2+}$, $V_{\mathrm{Li}}^{3+}$ and $V_{\mathrm{Li}}^{4+}$ ions act as extrinsic photorefractive centers that enhance the photorefractive properties of LN:V, as compared with CLN. In our recent paper, we reported that vanadium ions shift its occupation sites from Li sites in LN:V to the Nb sites with high ODR ion doping of magnesium ions in LN:V,Mg_6.0_ [26]. Here we suggest the same phenomena occur in the LN:V,Zr,Fe (LN1–LN3) crystals also. As it has Fe and Zr doping, therefore the Fe will preferably occupy and remain on Li sites due to its low valence, which can also be confirmed from the infrared OH absorption spectra, as no 3507 cm^−1^ exist for LN1-LN3 crystals, and from the UV absorption band peak enhancement at ~476 nm that shows the increase in ${\mathrm{Fe}}_{\mathrm{Li}}^{2+}$ donor sites with increased zirconium concentration resulting in the high diffraction efficiency. There is also a direct relationship of the Fe^2+^ to the current density $J\propto \left[{\mathrm{Fe}}^{2+}\right]$ that also confirms the enhancement of PR-time with an increase of Fe^2+^ concentration in LN:V,Zr,Fe crystals [41]. Additionally, the movement of the UV–Vis absorption edges toward the red with the increase in Zr concentration shows that Zr is already above the threshold concentration. The V ions which have comparatively high valence from Fe and Zr and is analogous to Nb and were at Nb_Li_ in the LN:V will be shifted from the Li sites to the Nb sites with this additional doping of Fe and Zr, forming defects of $V_{\mathrm{Nb}}^{2-/-}$ that act as new fast PR-centers. Therefore, the difference in the PR properties of LN:V,Zr,Fe crystals from those of LN:Zr,Fe and LN:Mg,Fe is visible from the defect structure, site occupancy, and location sites surplus of pentavalent vanadium ions.

Also, it is reported that, in LN:Fe, the charge transportation through polaron hopping of electron/holes is slow and is processed on Li-sites due to the presence of Nb_Li_ defects. It was suggested that in the absence of the antisites Nb, the hopping transport is shifted to the Nb sites and is much faster than chaotic Li-sites [42,43,44]. Therefore, doping V and Zr with Fe ions changes the hopping transport to Nb sites by reducing the Nb_Li_ sites and does not disturb the usual lithium site occupation of Fe and instead helps in eliminating the undesired intrinsic traps as shown in Figure 5b. This helps in enhancing the charge transport and response speed of LN and achieving a higher diffraction efficiency compared to other low valence dopants at 488 nm and 532 nm wavelengths.

Nevertheless, it is quite important to realize the relationship between the atomic as well as electronic configurations of the doping stability and the dopants. Furthermore, the understanding of doping stability in LN can better help us in the selection of the proper dopant necessary for the experimental conditions and applications.

## 5. Conclusions

In conclusion, we have grown various LN:V,Zr, and LN:V,Zr,Fe crystals by Czochralski method and studied their PR properties. The PR properties of LN:V,Zr,Fe crystals are enhanced tremendously compared with the LN:V, LN:Fe, and V and Fe co-doped LN crystals. The response time of LN:V,Zr_4.0_,Fe is shortened to 0.53 s with a high diffraction efficiency of 57%, and the sensitivity can reach 9.2 cm/J. From the above results and discussion, it is evident that both Zr- and Fe-doping have benignant effects on the PR properties of LN:V and the LN:V,Zr,Fe crystals can be considered as another potential material for 3D-holographic storage devices and dynamic holography applications.

## Figures and Tables

**Table 1:** Composition ratio of various iron, zirconium, and vanadium co-doped LN crystals.

Sample Symbol	Fe (wt.%)	Zr (mol%)	V (mol%)
LN:V,Zr_2.0_ (LN1)		2.0	0.1
LN:V,Zr_3.0_ (LN2)		3.0	0.1
LN:V,Zr_4.0_ (LN3)		4.0	0.1
LN:V,Zr_2.0_,Fe (LN4)	0.03	2.0	0.1
LN:V,Zr_3.0_,Fe (LN5)	0.03	3.0	0.1
LN:V,Zr_4.0_,Fe (LN6)	0.03	4.0	0.1
LN:V,Fe (LN7)	0.03		0.1

**Table 2:** The response times and diffraction efficiency of various co-doped and congruent LN crystals.

Crystals	LN:V,Zr_4.0_,Fe	LN:V,Zr_4.0_	LN:Zr,Fe,Mn	LN:Zr_5.0_,Fe	LN:Mg_6.0_,Fe	LN:Fe	LN:Bi,Mg_6.0_	CLN
τ_r_/s	0.53	1.1	0.95	2	15	120	0.17	180
η/%	57	2	55	42	15	69	18	0.6
	@488 nm this work	@488 nm this work	@532 nm [16]	@532 nm [15]	@488 nm [28]	@488 nm [21,28]	@488 nm [25]	@532 nm [21,25]
