# Correction: Saeed, S., et al. Enhancement of Photorefraction in Vanadium-Doped Lithium Niobate through Iron and Zirconium Co-Doping. Materials 2019, Vol. 12, 3143

**DOI:** 10.3390/ma13225299

**Published:** 2020-11-23

**Authors:** Shahzad Saeed, Hongde Liu, Liyun Xue, Dahuai Zheng, Shiguo Liu, Shaolin Chen, Yongfa Kong, Romano Rupp, Jingjun Xu

**Affiliations:** 1 The MOE Key Laboratory of Weak-Light Nonlinear Photonics, School of Physics and TEDA Institute of Applied Physics, Nankai University, Tianjin 300071, China; shehzadsaeed2003@yahoo.com (S.S.); 1120150044@mail.nankai.edu.cn (L.X.); dhzheng@nankai.edu.cn (D.Z.); nkliusg@nankai.edu.cn (S.L.); chenshaolin@nankai.edu.cn (S.C.); jjxu@nankai.edu.cn (J.X.); 2 Faculty of Physics, Vienna University, A-1090 Vienna, Austria; romano.rupp@univie.ac.at; 3 Department of Complex Matter, Jozef Stefan Institute, Jamova 39, SI-1000 Ljubljana, Slovenia

The authors wish to make the following corrections to this paper [1]. Due to mislabeling, replace:

**Table 1:** Composition ratio of various iron, zirconium, and vanadium co-doped LN crystals.

Sample Symbol	Fe (wt.%)	Zr (mol%)	V (mol%)
LN:V, Zr_2.0_ (LN1)		2.0	0.1
LN:V, Zr_3.0_ (LN2)		3.0	0.1
LN:V, Zr_4.0_ (LN3)		4.0	0.1
LN:V, Zr_2.0_, Fe (LN4)	0.03	2.0	0.1
LN:V, Zr_3.0_, Fe (LN5)	0.03	3.0	0.1
LN:V, Zr_4.0_, Fe (LN6)	0.03	4.0	0.1
LN:V, Fe (LN7)	0.03		0.1

with:

**Table 1:** Composition ratio of various iron, zirconium, and vanadium co-doped LN crystals.

Sample Symbol	Fe (wt.%)	Zr (mol%)	V (mol%)
LN:V,Zr_2.0_,Fe (LN1)	0.03	2.0	0.1
LN:V,Zr_3.0_,Fe (LN2)	0.03	3.0	0.1
LN:V,Zr_4.0_,Fe (LN3)	0.03	4.0	0.1
LN:V,Zr_2.0_ (LN4)		2.0	0.1
LN:V,Zr_3.0_ (LN5)		3.0	0.1
LN:V,Zr_4.0_ (LN6)		4.0	0.1
LN:V,Fe (LN7)	0.03		0.1
LN:V (LN8)			0.1

The authors would like to apologize for any inconvenience caused to the readers by these changes.
